# Analyzing blank cutting edge efficiency associated with the adoption of microblade technology: A case study from Tolbor-17, Mongolia

**DOI:** 10.1371/journal.pone.0305136

**Published:** 2024-08-16

**Authors:** Corey L. Johnson, Tsedendorj Bolorbat, Mark N. Grote, Clea H. Paine, Guunii Lkhundev, Davaakhuu Odsuren, Masami Izuho, Byambaa Gunchinsuren, Nicolas Zwyns

**Affiliations:** 1 Department of Anthropology, University of California Davis, Davis, CA, United States of America; 2 Institute of Archaeology, Mongolian Academy of Sciences, Ulaanbaatar, Mongolia; 3 Archaeology Institute, University of the Highlands and Islands, Kirkwall, United Kingdom; 4 Department of History, Mongolian National University of Education, Ulaanbaatar, Mongolia; 5 Faculty of Humanities and Social Sciences, Tokyo Metropolitan University, Tokyo, Japan; 6 Department of Human Evolution, Max Planck Institute for Evolutionary Anthropology, Leipzig, Germany; The University of Tulsa, UNITED STATES OF AMERICA

## Abstract

The phenomenon of lithic miniaturization during the Late Pleistocene at times coincided with increased artifact standardization and cutting edge efficiency–likely reflecting the use of small, sharp artifacts as interchangeable inserts for composite cutting tools and hunting weapons. During Marine Isotope Stage 2, Upper Paleolithic toolmakers in northern East Asia specifically used pressure techniques to make small, highly standardized lithic artifacts called microblades. However, little is currently known about how microblades affected the cutting edge efficiency of the toolkits they were a part of. We applied three methods of analyzing cutting edge efficiency to two Upper Paleolithic assemblages recently excavated from Tolbor-17, Mongolia, that document the periods before and after the introduction of microblade technology to the Tolbor Valley. A model incorporating allometric relationships between blank cutting edge length and mass suggests no difference in efficiency between the two periods, while two more conventional approaches both indicate a significant increase. The potential for improved cutting edge efficiency is only observed when the microblade sample is artificially inflated via simulation. Our results highlight challenges related to detecting and interpreting archaeological differences in cutting edge efficiency at the assemblage level.

## Introduction

Blank production (i.e. the making of lithic flakes, blades, bladelets, etc.) is thought to have emerged as an intentional technological behavior within the hominin lineage in part due to the capacity for a blank to be used in a subsistence based cutting task [1–10]. Subsequent changes in the procedural steps of blank production (methods) and physical means of blank formation (techniques) [11] observed within the lithic archaeological record are at times thought to have resulted in corresponding effects on blank cutting edge efficiency ‐ often defined as the length of a blank’s cutting edge relative to its volume [12–18]. From this perspective, as changes in hominin physiology [19–21] and paleoecology [9, 22, 23] occurred, blank production methods and techniques which economized the amount of cutting edge available to meet changing energy demands [24] would have represented a fitness benefit to individuals that acquired them [17]. In other words, being able to make tools with high blank cutting edge efficiency would have increased the biological fitness of toolmakers because they would have had more energy to devote to other tasks.

Previous research suggests that blank cutting edge efficiency, and the variability around this parameter, gradually increased throughout the Pleistocene [16–18]. However, it remains unclear exactly how different blank production strategies contributed to this trend, as experimental studies find little difference in cutting edge efficiency between systems of flake and blade production like those employed by lithic toolmakers from the Early to Late Pleistocene [13, 16, 25, 26]. Nonetheless, archaeological and experimental results suggest that blank standardization and cutting edge efficiency improved during the Late Pleistocene with the development of methods of blank miniaturization [18, 27] and pressure techniques of blank production [16, 27, 28] ‐ with Late Upper Paleolithic microblade technologies notably representing a combination of both of these technical behaviors [29].

Microblades are small, elongated, very thin blanks often exhibiting a straight lateral profile, narrow, parallel edges, a punctiform platform, and a small compact bulb [29]. These traits are largely the result of the pressure techniques used to produce them [30], which involved the application of a static force to the edge of a core’s striking platform using the pointed tip of a pressure crutch, or tine, until detachment via the mechanics of blank formation [31–34]. The small surface area of the tip of the tine would result in a laterally narrow blank platform, which in controlled experiments has been shown to be foundational to the production of narrow blanks [35, 36]. Static loading would also allow for precise control over platform depth and angle of force, parameters which have been shown to predetermine blank size and shape [15, 37–39]. Finally, methods of microblade core shaping and maintenance [40, 41] would have allowed for control over the morphology of the core’s flaking surface and exterior platform angle, parameters which have been shown to have important effects on blank shape ‐ particularly blank thickness and elongation [15, 37, 42].

The development of microblade technology is generally thought to have been driven by a need to increase artifact standardization, utility, and transportability during the Last Glacial Maximum (LGM) climatic episode of Marine Isotope Stage 2 (MIS 2) [27, 43–50]. For humans subsisting in the northern latitudes of East Asia, where microblade technologies first appeared and spread [29], the LGM would have triggered a significant conflict between the availability of food and lithic raw material on the landscape. With the onset of colder climates during the LGM, the carrying capacity of these northern environments would have decreased [51], causing the large-bodied mammals that inhabited them to become more widely distributed [46, 49]. This in turn would have required human groups that relied on these animals as sources of food and materials to increase their mobility and regularly operate further away from geographically fixed lithic raw material sources [44–46, 47, 50].

From this perspective, microblades are thought to have been a technological solution to an ecological dilemma, as methods and techniques underlying microblade production effectively maximized the number of small, standardized blanks that could be made from more easily transportable cores [16, 27, 43, 48, 50]. These blanks were possibly used in hand as small cutting tools or inserted into the mortises of composite cutting tools [47] and hunting weapons [27, 46, 49] ‐ the latter suggested by their high degree of standardization and technological investment [27, 52]. However, it is unclear if microblades increased the cutting edge efficiency of the lithic toolkits they were a part of, as little work has attempted to measure their impact on this parameter (though see Elston and Brantingham’s [43] rough comparison of microblade and biface cutting edge efficiency; and studies on pressure macro-blade cutting edge efficiency [16, 28]). To address this question, we first selected two lithic assemblages recently excavated from the Upper Paleolithic site of Tolbor-17, Mongolia, which document the periods before and after the adoption of microblade technology in the Tolbor Valley.

### Tolbor-17

**Fig 1:**
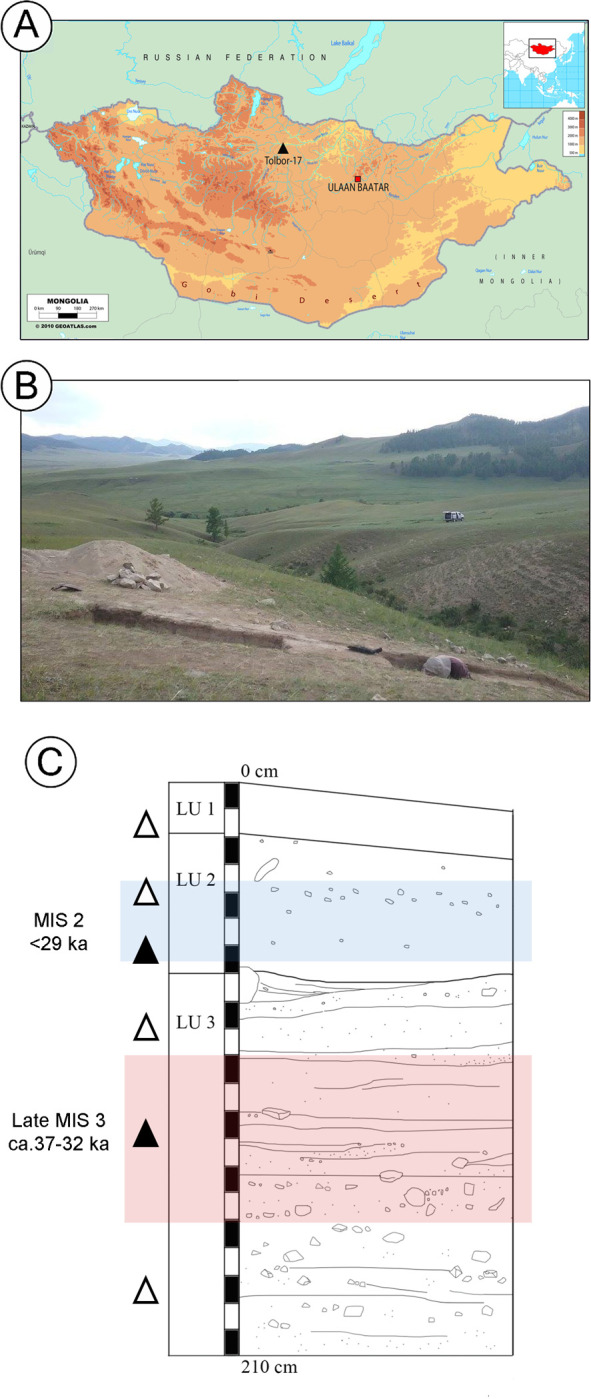
Location and stratigraphy of T17. A) Elevation map showing geographic location of T17; B) South facing view of T17 during the 2017 excavation; C) Schematic section drawing of the stratigraphy at T17 with the approximate location of artifact accumulations and denser artifact concentrations indicated by open (△) and closed (▲) triangles, respectively. Colored bands indicate the vertical extent of the LU2 and LU3 lithic samples. Map modified after Geo-atlas and Gallo et al. [54].

Tolbor-17 (T17) is an open-air site located in northern Mongolia along a low altitude pass above the Ikh-Tolborin-Gol (‘Great Tolbor River’ in English), a tributary of the Selenga River basin that flows into Lake Baikal to the north (Fig 1) [53, 54]. The site was originally identified by A. Tabarev and S. Gladyshev who conducted the excavation of two test pits in 2010, and suggested a MIS 3 occupation of the site based on the recovered lithic assemblage and an obtained radiocarbon date [55, 56]. Full-scale excavation at T17 began in 2017, initially consisting of two new 2x1 m test pits with, as of 2019, the excavated surface having been expanded to ca. 18 m^2^. During each stage of excavation archaeological material >2 cm was piece plotted, and excavated sediment was dry sieved using 4 mm and 2 mm mesh screens.

Three main lithological units are identified at T17. These include the LU1 Holocene soil complex, the LU2 loess and loess-like deposits, and the LU3 laminar silt with gravel and cobbles. Depositional variability within LU3 is currently under investigation, with the eventual possibility of further stratigraphic subdivision deeper within the unit. Our study samples two artifact assemblages, one from LU3 and one from LU2 (Fig 1C), as these units document the period before and after the appearance of microblade technology in the valley. The LU3 assemblage contains Early Upper Paleolithic (EUP) blade and bladelet technologies as well as diagnostic EUP tool types such as endscrapers and perforators. Previous test excavation at T17 dated a level that corresponds with the LU3 sample to 33–34 ka cal. BP [57, 58]. Stratigraphic correlation with other Upper Paleolithic sites in the Tolbor Valley [53, 59] also places the material from LU3 in the later period of MIS 3, or between ca. 37–32 ka cal. BP. The LU2 assemblage contains microblades and microblade cores characteristic of the Late Upper Paleolithic (LUP). The equivalent unit to LU2 at other Tolbor sites is either younger than ca. 29 ka cal. BP or yields an MIS 2 age [53, 59].

Other Upper Paleolithic sites discovered in the Tolbor Valley besides T17, such as T4, T15, T16, and T21, also document periodic episodes of human occupation in the region ‐ beginning with the Initial Upper Paleolithic (IUP) and continuing to the end of the Pleistocene [53–61]. The appearance of the IUP in the Eurasian steppe coincides with the earliest fossil evidence of *Homo sapiens* in the region, suggesting that the IUP documents the expansion of our species eastward across North-Central Asia ca. 45 ka [53, 62–66]. The persistence of *H*. *sapiens* in the fossil record of Mongolia [67] coinciding with the archaeological record preserved at T17, further suggests that the Upper Paleolithic assemblages within the site were formed by members of our species as well.

To investigate the cutting edge efficiency of lithic assemblages in the Tolbor Valley during the transition from MIS 3 to MIS 2, we ask: 1) whether the assemblage sampled from LU2 has a higher signature of blank cutting edge efficiency than the LU3 assemblage and 2) if any difference in cutting edge efficiency observed between the assemblages can be attributed to microblade production.

### Measuring blank cutting edge efficiency

There are multiple methods of measuring blank cutting edge efficiency, each with their own built-in assumptions, strengths, and weaknesses [68]. While an assessment of each method used in the literature is beyond the scope of this study, we provide a brief critical review of those that are most relevant.

In many studies concerning blank cutting edge efficiency, ratios of blank perimeter length to mass (as a proxy for volume) are commonly used. For example, the equation:

PerimeterLength/Mass

is in part used by Braun and Harris [12]. Similarly, Mackay [14] proxies blank perimeter length using three linear measurements, producing the form:

$$\left(Max\;Length+Max\;Width+Max\;Dimension\right)/Mass$$

which has been found to be closely correlated with the variable used by Braun and Harris [see also 68]. Mackay’s proxy is the most widely used in the literature as it is easy to measure and calculate, making comparisons between large datasets relatively straightforward [e.g. 10, 69–74]. It should be noted however, that both equations use, or proxy, measurements of blank *perimeter* to estimate cutting edge efficiency [12, 14], regardless of whether the entire perimeter of said blank is sharp enough to perform a cutting task (termed here as its “cutting edge length”) [75, 76].

Sometimes the scaling exponent between blank cutting edge length and mass is assumed to be 1/3, which implies that an increase in blank mass is analogous to an increase in volume (given that the raw material has a uniform density). For example, Stout et al. [77] adjust Braun and Harris’ [12] equation with the form:

$$Cutting\;edge\;Length/Mass^{1/3}$$

to account for the nonlinear relationship between cutting edge and mass. Similarly, Morgan et al. [78] use the form:

$$Cutting\;edge\;Length/Mass^{1/3}*\left(1-exp\;\left[-0.31*\left(Max\;Dimension–1.81\right)\right]\right)$$

to also account for the influence of a blank’s size on its cutting edge efficiency, rewarding blanks for having high cutting edge efficiency and penalizing them for being small. Stout et al. found that these two equations were highly correlated and produced qualitatively similar results [77]. Notably both equations also specifically measure blank cutting edge length, rather than the entire perimeter of a blank.

Other times the scaling relationship assumed in cutting edge analyses is not between blank shape and mass. For example, Režek et al. [17] use the dimensionless form:

$$\left(Length*Width\right)/Thickness^{2}.$$

In their analysis, blank volume is represented by blank thickness, which is “squared to bring its variance and the variance of blank surface area to the same scale” [17]. Like Mackay’s [14] estimate, this approach is attractive for dealing with large data sets from multiple sites, as the measurements used to proxy blank surface area are easy to collect and extract from the literature. Also similar is the fact that Režek et al.’s [17] method uses a proxy for blank perimeter, instead of blank cutting edge length.

An alternative approach to those outlined above does not impose a particular scaling between variables, and instead discovers what the scaling is from the artifacts themselves. For example, blank cutting edge length and mass may be projected into a log-log Cartesian plane [e.g. 12, 79]. Such an approach implies an allometric relationship between cutting edge length and mass [68] in the form of a power-law:

$$Cutting\;edge=\mu^{*}Mass^{\beta }.$$

**Fig 2:**
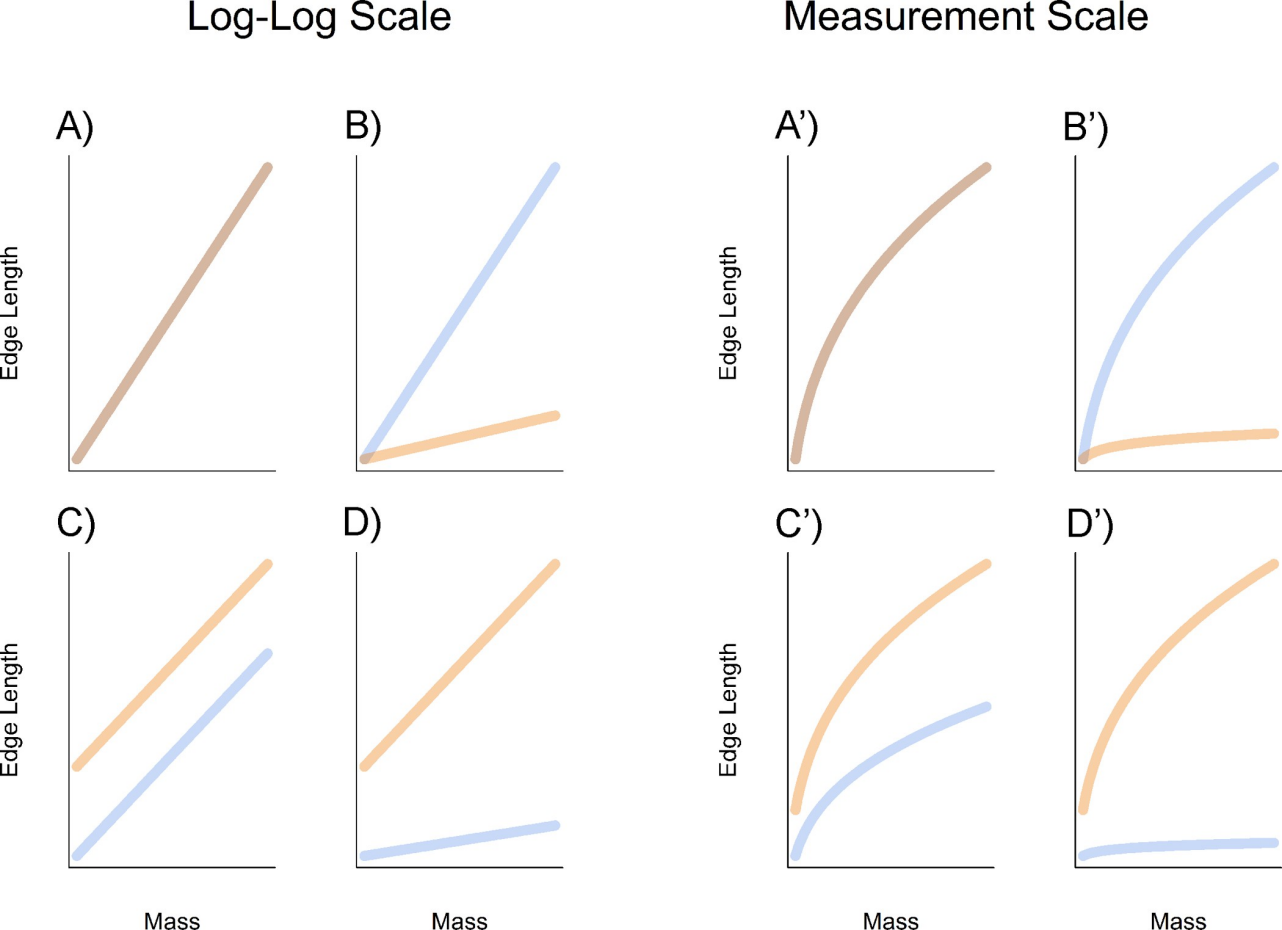
Variations in the allometric relationship between cutting edge length and mass in the measurement scale, expressed as differences in intercepts and/or slopes in the log-log scale. **A and A’)** no differences in slopes or intercepts; **B and B’)** different slopes with shared intercept; **C and C’)** different intercepts with shared slope; **D and D’)** slopes and intercepts both different.

Here there are two free parameters: μ, the allometric coefficient; and β, the allometric exponent [80]; which characterize different aspects of cutting edge efficiency. Braun and Harris [12] hint at an allometric relationship between cutting edge length and mass, as “two measures of size that increase at different rates”. The change in allometry presented by Braun and Harris [12: Fig 9] as an increase in cutting edge efficiency is illustrated here by panels C and C’ in Fig 2. As we show in Fig 2, other variations in allometry, also indicating changes in cutting edge efficiency, are possible.

With these details in mind, we adapted Braun and Harris’ [12; see also 79] log-log approach into a statistical model (described below) to test for changes in cutting edge efficiency corresponding with the appearance of microblades at T17. We then compared the results with those of the more widely used methods published by Mackay [14] and Režek et al [17].

## Materials and methods

### Qualitative and quantitative data collection

**Fig 3:**
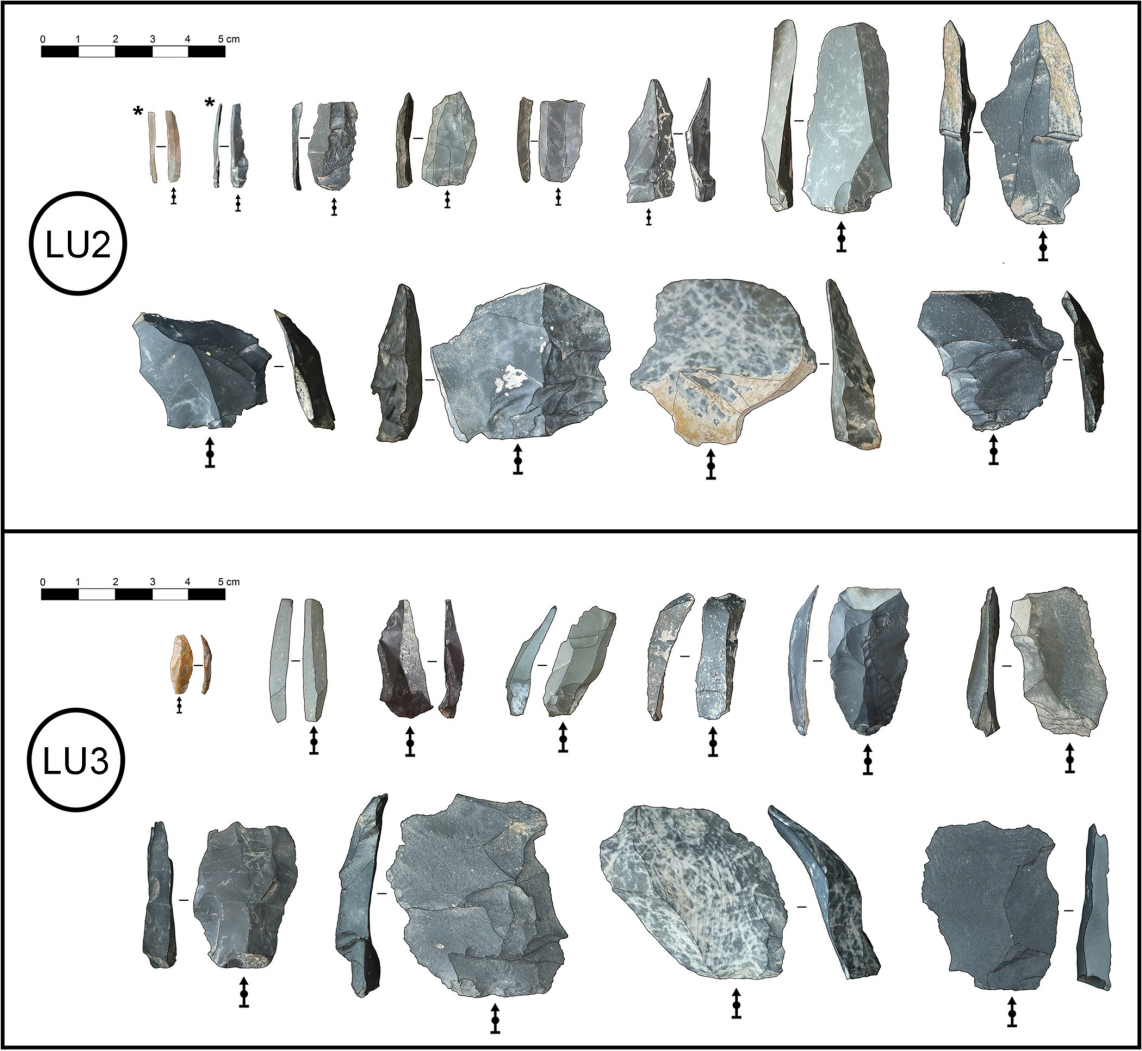
Examples of blanks from LU2 and LU3 used in the study. Asterix denotes microblades.

Lithic data collected from the LU3 and LU2 sample assemblages for this study included: 1) blank completeness; 2) blank length, width, thickness, and mass; 3) blank production method and technique; 4) the presence or absence of retouch; and 5) cutting edge length [81]. All complete, unretouched blanks were included in this study given that incomplete or retouched blanks are missing part of their initial mass and cutting edge length. Complete blanks are defined here as blanks that preserve a distal termination, mesial edges, and proximal portion including the platform. Blanks were not disqualified from our analysis due to their role within a reduction sequence, whether predetermined or predetermining [81], as any blank with a cutting edge could potentially have been used in a subsistence based cutting task [15, 18, 77, 82]. The total sample consisted of 433 blanks from LU3, and 156 blanks from LU2 ‐ including 10 microblades (Fig 3 and Table A in S1 File). All artifacts studied are curated at the Institute of Archaeology branch of the Mongolian Academy of Sciences in Ulaanbaatar. No permits were required for the described study, which complied with all relevant regulations (S1 Checklist).

**Fig 4:**
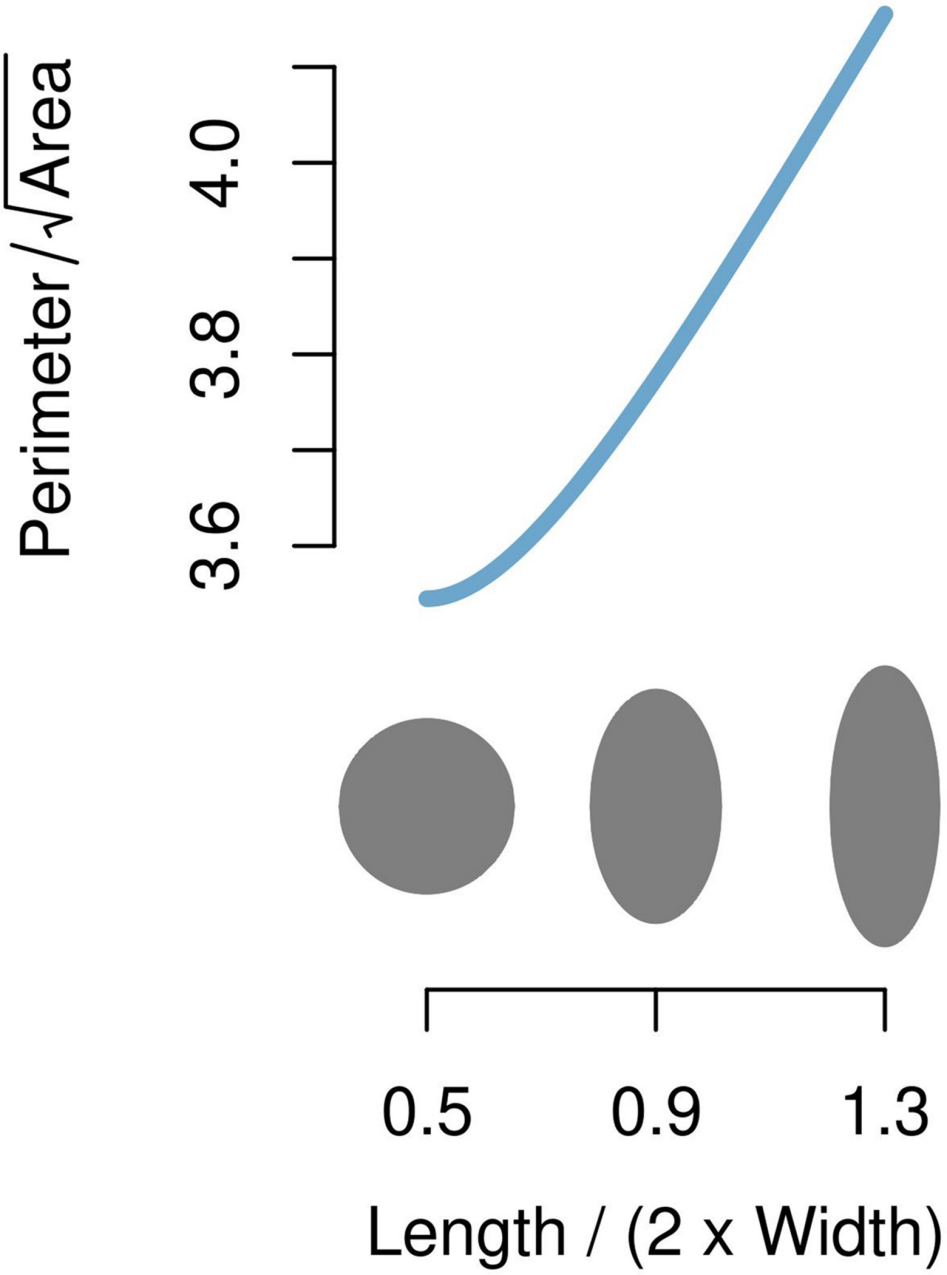
Relationship between the perimeter of an elliptical flake and the ratio Length / (2 x Width). The perimeter is given by Ramanujan’s second approximation [83: equation 50] and is scaled by the square-root of the ellipse’s area. The ellipses shown in grey have identical areas and correspond to Length / (2 x Width) ratios 0.5, 0.9, and 1.3 as depicted below the curve. The scaled perimeter increases as the ellipse becomes more elongated, suggesting greater cutting edge length per area for more blade-like blanks.

Metric data for the length and width of each blank were collected using the ‘box method’, as described by Dogandžić et al [68: Fig 3B], which records the length and width dimensions of a blank relative to its axis of percussion, or technological axis. These measurements also allow for a comparison of the results of our model as described below with the more commonly used methods of analyzing cutting edge efficiency developed by Mackay [14] and Režek et al. [17]. We note here that we risk tautological reasoning if we compare blank cutting edge efficiency across blank type categories defined by different length to width relationships, such as blades and flakes [81]. For example, idealized elliptically shaped blanks, having different length to width ratios, will have deterministically different perimeter lengths, and therefore potentially more cutting edge, per area (Fig 4). More elongated (i.e. blade-like) blanks will have greater perimeter per area than less elongated (i.e. flake-like) blanks [see also 26]. Naïve comparison of cutting edge length solely between blades and flakes may therefore fail to account for these “built in” differences. Some studies presumably combine all blank types in cutting edge analyses for this reason when more than one blank type defined by length to width relationships exists in the sample [e.g. 14, 17], and we also follow this approach here.

As in some studies [e.g. 18, 25, 77, 78, 84, 85], our definition of cutting edge length only included the segments of a blank’s perimeter that exhibit a sharp edge between its dorsal and ventral surface. Specifically, edge lengths exhibiting an angle <50° for blanks that are <40 mm, and an angle that that is <70° for blanks that are >40 mm. The definition of what constitutes a viable cutting edge used here is based on what has been experimentally observed as the threshold of a stone artifact edge to optimally cut through organic material [75, 76].

All relevant data were collected into an E4-MSAccess database (oldstoneage.com). For metric data, a standard digital caliper, digital scale, and a metric sewing tape measure were used. The sewing tape measure was used to record the length of a blank’s cutting edge to the nearest 5 mm to minimize precision error following the logic laid out by Dibble and Bernard [86].

### Statistical modeling

We test for differences in cutting edge efficiency between the LU3 and LU2 lithic assemblages using a statistical model which compares all blanks within the two assemblages. We fit linear regression models for Log10 cutting edge length, the dependent variable, with Log10 mass and assemblage (LU3 or LU2) as independent variables, like Braun and Harris [12] and Braun [79]. A factorial ’Analysis of Covariance’ model allows for the possibility that slopes and intercepts are unique for each group of blanks. Therefore, for blank *b*, in assemblage *a*, the model has the form:

$${\text{log}}_{10}Cutting\;edge\;Length_{b,a}{\sim \text{log}}_{10}Mass_{b,a}*Assemblage_{a}+\varepsilon_{b,a}.$$ (1)

Because heavier, and therefore larger, blanks offer the potential for greater variability in cutting edge length, we apply regression weights of the form:

$$w_{b,a}=1/{\text{log}}_{10}Mass_{b,a}$$ (2)

implying that the variance of *ε* is proportional to log_10_ *Mass* [87: Section 5.1.1].

Some complete, unretouched blanks included in this study did not have any segment with a useful cutting edge angle and were consequentially recorded as having 0 mm of useful cutting edge length. Blanks with zero cutting edge length are shown in graphical displays but are not included in the statistical models because of their small sample size (n = 10 across both assemblages) and unsuitability for log transformation. Larger numbers of blanks having zero cutting edge length could, in principle, allow for a model that compares both failure rate and cutting edge efficiency across levels [e.g. 88: the "Hurdle Model"].

For comparison with the statistical model, we also applied the approaches used by Mackay [14] and Režek et al. [17] to test for significant differences in cutting edge efficiency between the assemblages. In response to a reviewer’s feedback, we carried out additional sensitivity checks to explore more fully the consequences of using direct or proxy measurements of blank cutting edge length, in combination with inferred or imposed allometry (S1 File).

### Simulated microblade sample

Although the microblade sample from T17 is relatively small (n = 10), the additional presence of diagnostic microblade fragments and microblade cores within the LU2 assemblage indicates that microblade production was one of the primary technological activities taking place on site. This potential recovery-bias of microblades at T17 may be due to several factors, including:

- The currently exposed area of the excavation relative to the true extent of the site, and the possibility of a structured use of space by its residents
- The preferential washing away of microblades during site formation due to their small size and light weight
- The preferential transport of microblades away from T17 by people living at the site for logistical use elsewhere in and around the Tolbor Valley

For this reason, we simulated additional microblade cutting edge length and mass values to better compare their allometric scaling with that of the non-microblade archaeological sample from T17. The simulations proceeded as follows: 1) we fit a log-log model of cutting edge length per unit of mass, similar to Eqs 1 and 2, to the archaeological microblade sample from LU2 (n = 10); 2) we simulated microblade mass by generating Normally-distributed variables having a mean equal to the average of the observed microblade mass after log10 transformation; 3) we simulated cutting edge lengths for the virtual samples from the fitted microblade model; and 4) we varied the number of simulated microblades based on frequencies reported from three other sites in East Asia dating to the MIS 2.

The sites we used to inform our simulated microblade samples were selected to show three different scenarios of preservation frequency. To this end, we used the frequency of complete microblades and proximal fragments (which together represent a minimum number of individual artifacts) reported from: 1) Kovrizhka IV (Level 6; squares 21, 16, and 11) located in northern Cisbaikal, Russia (n = 33) [89]; 2) Xishahe (Layer 3A) located in northern China (n = 107) [90]; and 3) the Kashiwadai 1 site located in Hokkaido, Japan (n = 275) [91].

Data analysis was performed in R [92] with the addition of the libraries Epi [93] and Scales [94]. All data and code used to perform the analysis herein can be found in S1 Data and S2 File.

## Results

**Fig 5:**
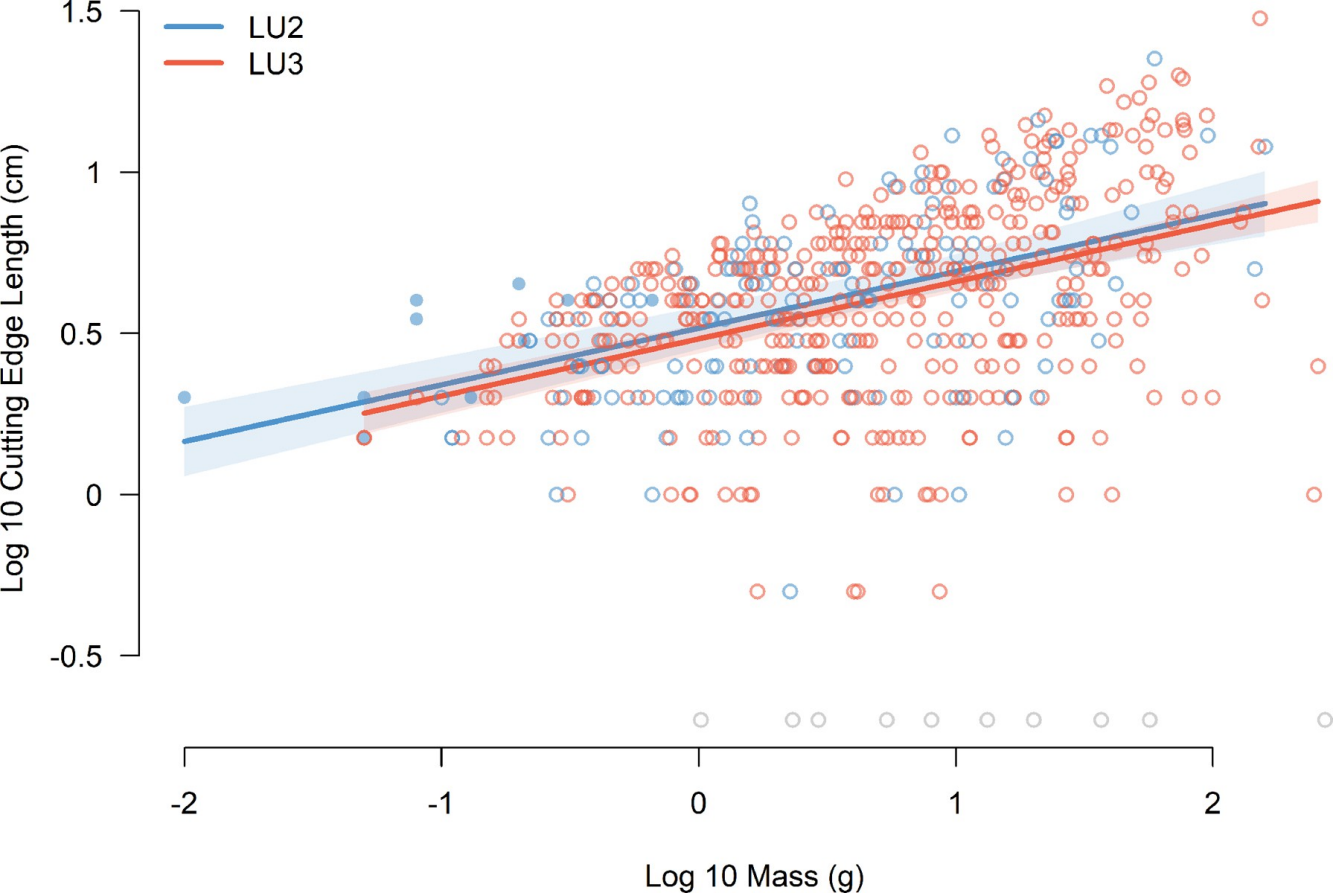
Graphical display of the fitted model comparing all complete blanks from LU3 and LU2. Blanks with zero-cutting edge are shown in grey and are positioned along the lower edge of the scatter plot, just above their Log 10 mass. Microblades from LU2 are indicated by closed circles (●).

Our statistical model found little difference in cutting edge efficiency between the lithic assemblages of LU3 and LU2 (Fig 5). Cutting edge length increases with blank mass in a predictable way in both samples; however a difference in cutting edge efficiency between the two levels, which would be indicated in the log-log plot by differences in intercepts, slopes, or both (Fig 2), is not supported. Though the LU2 central line is slightly above that for LU3, suggesting a slight increase in efficiency, their lines are closely spaced and parallel, and their confidence bands substantially overlap. A cone shaped scatter, widening as blank mass increases, affirms that the variance in cutting edge length also increases with blank mass.

The estimated slopes from these models, reflecting the allometry exponents for the LU3 and LU2 samples [80], are typically less than 1/3. A slope of 1/3 is what would be expected from a purely geometric relationship of a blank’s cutting edge (a linear measurement) with its mass (a volumetric measurement) [77, 78]. The slopes for the LU2 and LU3 lines of central tendency are each 0.18, and neither of their 95% confidence intervals contains the value 1/3.

**Fig 6:**
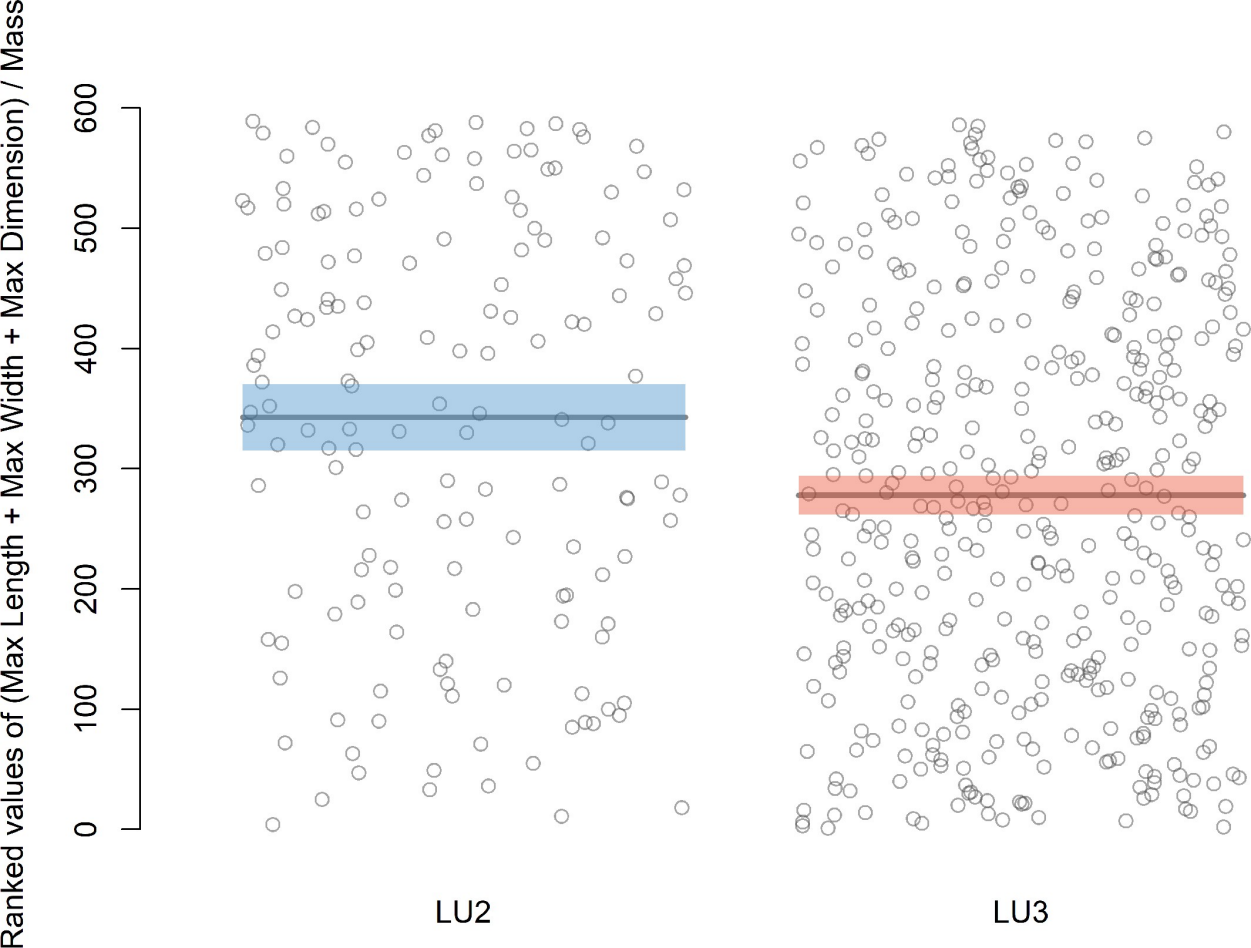
Graphical display of ranked values of the cutting edge efficiency statistic in Mackay [14] for each blank in the LU2 and LU3 assemblages. Means of ranked values are shown as horizontal lines, with shaded bands indicating two standard-error intervals around the means. Means and standard errors are shown here only for visualization purposes and do not reflect the calculation of the Wilcoxon statistic explicitly.

**Fig 7:**
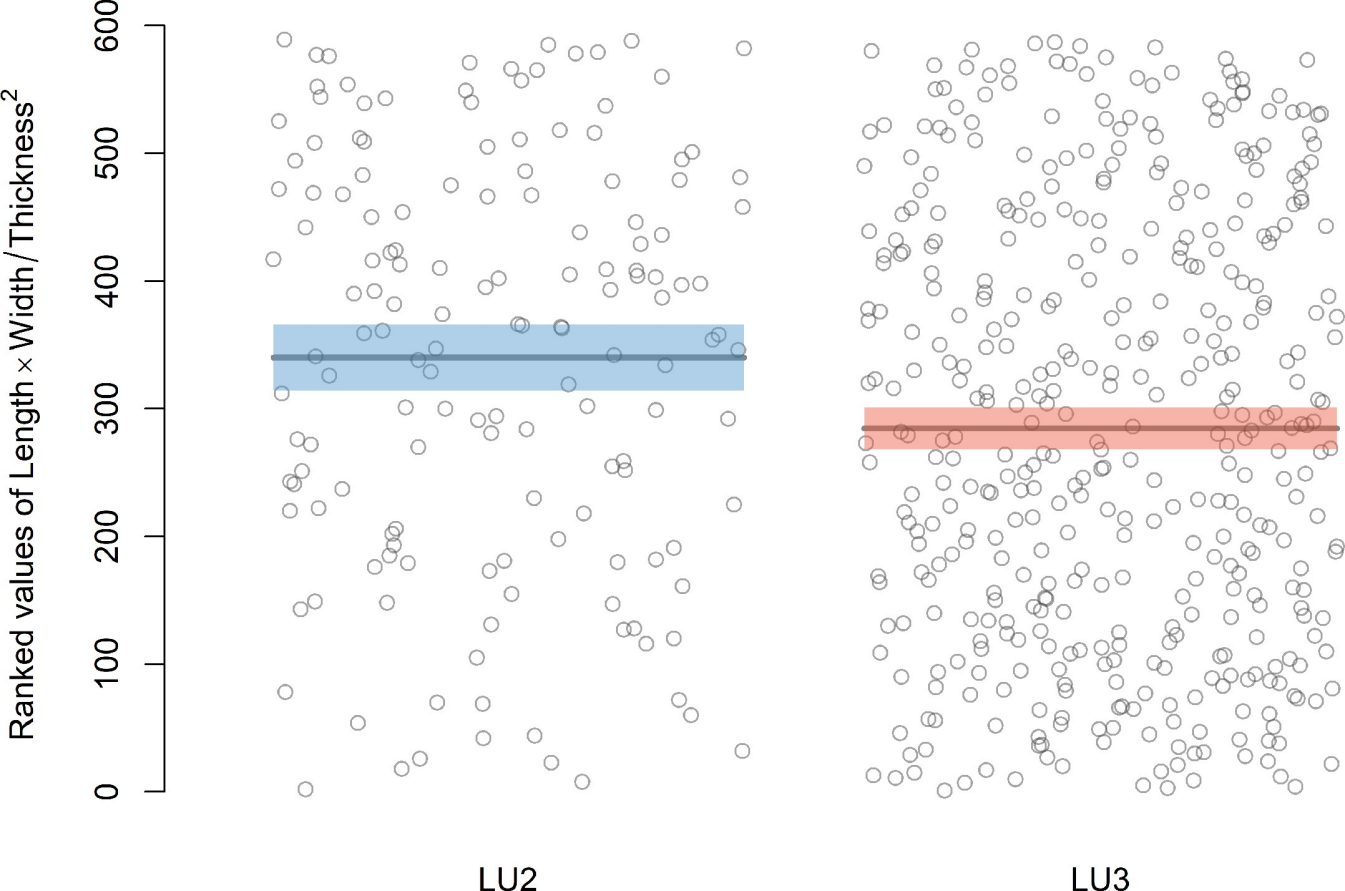
Graphical display of the ranked values of the cutting edge efficiency statistic in Režek et al. [17] for each blank in the LU2 and LU3 assemblages. Means of ranked values are shown as horizontal lines, with shaded bands indicating two standard-error intervals around the means. Means and standard errors are shown here only for visualization purposes and do not reflect the calculation of the Wilcoxon statistic explicitly.

Comparisons of cutting edge efficiency based on variables published by Mackay [14] and Režek et al. [17] suggest significant differences between the LU3 and LU2 assemblages. A Wilcoxon ranked sum test based on Mackay’s [14] equation using all blank types in each sample returns the test statistic W = 40020 and a p-value of <0.001 (Fig 6), indicating that the blanks in LU2 are more efficient than the blanks in LU3. Similarly, a Wilcoxon rank sum test based on Režek et al.’s [17] equation returns the test statistic W = 38614 and a p-value of <0.001 (Fig 7), also indicating that the blanks in LU2 are more efficient. We applied the rank sum test in both cases for standardization purposes, although a two sample t test is suggested originally by Mackay [14].

Our sensitivity checks for proxied cutting edge length and inferred allometry are reported in the supplementary materials (S1 File). When proxied measurements for blank cutting edge length are used in a log-log model with blank mass as a predictor, allowing the allometry to be inferred, results are variable (Fig A in S1 File). Use of the Mackay proxy suggests a significant difference between the assemblages, while the Režek et al. proxy does not. When direct measurements for blank cutting edge length are used in equations that impose a certain allometry (Mackay [14], and Režek et al. [17], respectively), both methods suggest significant differences between assemblages (Figs B and C in S1 File).

**Fig 8:**
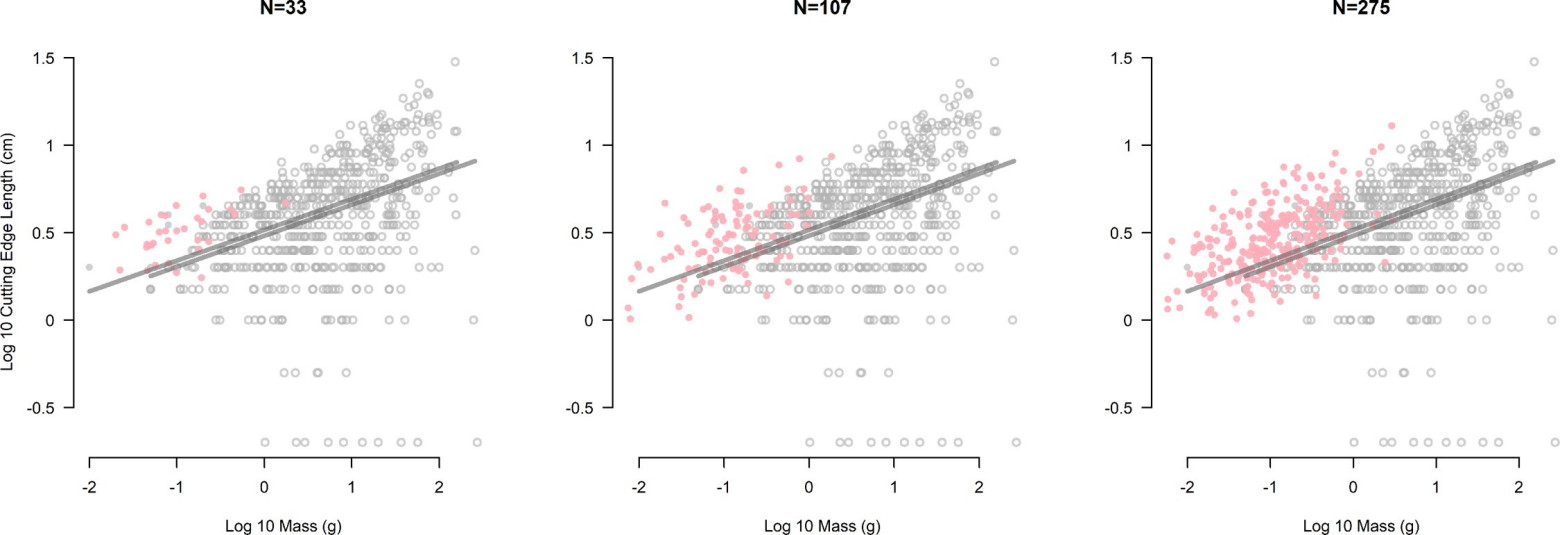
Graphical display of simulation experiments using microblade data from LU2. Simulated sample sizes displayed from left to right are based on microblade frequencies reported from Kovrizhka IV [89], Xishahe [90], and Kashiwadai 1 [91], respectively. In each display, simulated microblades are indicated by pink closed circles (●), and archaeological materials from LU3 and LU2 are shown in grey while following the conventions described in Fig 5. The lines of central tendency for LU3 and LU2 are shown in each panel unmodified from Fig 5: in particular, the central line for LU2 was not re-estimated for the simulated microblades.

Finally, when larger groups of microblades are simulated using data from LU2, we see them appear as a distinct population relative to the rest of the archaeological blanks (Fig 8). The cutting edge to mass ratios of these virtual microblades exceed those of the other archaeological blanks of similar size, indicating greater efficiency and a unique scaling of cutting edge to mass.

## Discussion

### Scaling relationships matter

While the adoption of methods and techniques underlying microblade production during MIS 2 may have a been a behavioral response to conditions which in part favored increasing blank standardization [27] and cutting edge efficiency [17, 18, 28, 43], our primary model finds little archaeological evidence for the latter at T17 (Fig 5). Our approach uses two free parameters which characterize cutting edge efficiency [12] (Fig 2). This characterization does not assume a particular scaling relationship between cutting edge length and mass, but instead discovers what the scaling is from the lithic sample under investigation. This is crucial, as the results of our simulations suggest that microblades have a different scaling relationship between cutting edge length and mass than blanks made using percussion techniques (Fig 8) ‐ likely due to the influence that pressure techniques have on blank shape and size [27, 28, 95].

**Fig 9:**
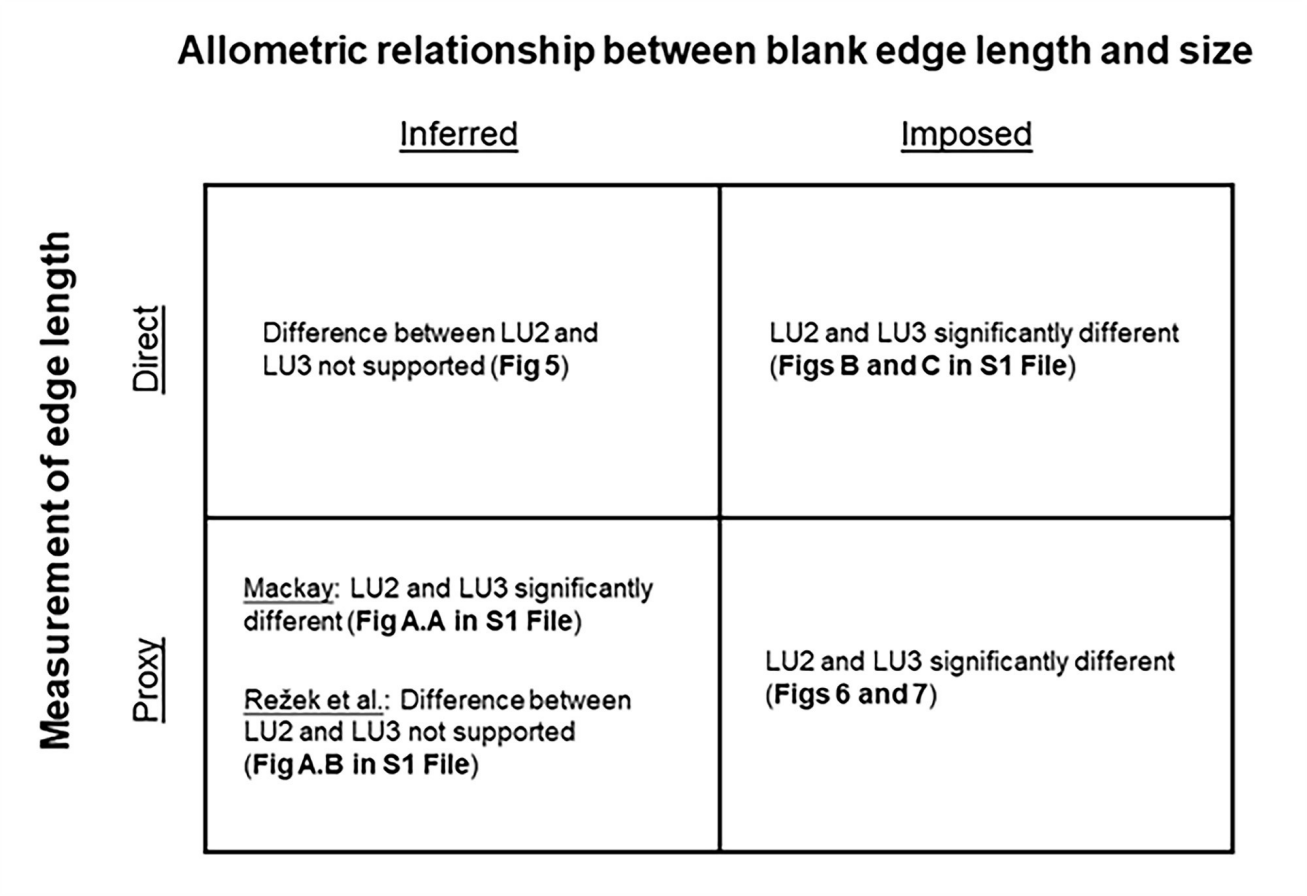
Sensitivity of results to different combinations of 1) the method of cutting edge length measurement (direct, and proxy [14, 17]); and 2) statistical approaches that infer, or impose [14, 17], allometry between blank edge length and size.

Our sensitivity checks found that proxied measurements for cutting edge length following Mackay [14] and Režek et al. [17], substituted for direct measures in our primary model (Eq 1), yield conflicting results regarding differences between LU3 and LU2 (Fig 9; Fig A in S1 File). While it may be tempting to suggest that proxy measurements perform reasonably well, at least in some cases, on principle direct measurements of cutting edge length should be used whenever possible. Finally, we note that proxy measurements of blank cutting edge length are not able to identify blanks which have edges that are too dull to perform a cutting task.

While the primary model found no statistical difference in cutting edge efficiency between the LU3 and LU2 samples, methods which assume particular scaling relationships did (Figs 6 and 7). The latter findings hold whether direct or proxied measurements of cutting edge length are used (Fig 9; Figs B and C in S1 File). Additionally, we did not find an allometry exponent of 1/3 in our model for either assemblage, implied in other analyses [77, 78]. Until we can better understand the cause(s) of these discrepancies we caution against making assumptions about how cutting edge length scales with mass, as doing so will have consequences for accurately characterizing blank cutting edge efficiency when comparing archaeological assemblages that contain blanks of various morphologies (S1 File) [see also 68].

At first glance our results seem to subvert expectations of a continuing trend of increasing cutting edge efficiency during the Upper Paleolithic related to the development of novel methods of lithic miniaturization and the use of pressure techniques [16–18, 27, 28, 43, 95]. However, they might be better understood as an example of the increased variability around this parameter also observed during the Late Pleistocene [17]. From this perspective the situation at T17 may be that, within the environmental contexts of the region, microblades were not made to increase blank cutting edge efficiency at the assemblage level between the EUP and LUP. The appearance of microblade technologies at T17 may therefore reflect the economization of other inter-related parameters of technological efficiency, such as raw material selection, artifact maintenance, artifact standardization, or artifact transportability [17, 27, 28].

### Assemblage level assumptions

Yet, that cutting edge efficiency did not change at the assemblage level with the adoption of microblade technology at T17 does not necessarily mean that selection pressures on increasing this parameter of lithic toolkit efficiency were absent. Selection pressures on increasing blank cutting efficiency might not always result in adaptation of the entire blank assemblage, instead only affecting the efficiency of certain blanks within a reduction system. For example, the development of microblades may be in part due to selective pressures which favored their increased cutting edge efficiency, yet the impact this had at the assemblage level may have been offset by the presence of relatively less efficient technical blanks needed to shape and maintain microblade cores [40, 41].

Such a scenario invites a reevaluation of the basic assumptions of blank cutting edge analyses ‐ particularly whether cutting edge efficiency at the assemblage level is a meaningful measure of change in the adaptive behaviors of Plio-Pleistocene hominins. While this may be the case when each blank in a reduction sequence is an end-product, made with the intention of being used in a cutting task [e.g. 12, 77], it may not be so for technological systems involving the production of different types of predetermining and predetermined blanks. For example, a core reduction system adapted to produce a blank of a specific shape and size, with a relatively higher cutting edge efficiency, may be equally or even less efficient as other, more generic core reduction systems when all blanks, including byproducts, from each system are considered together [e.g. 16, 26].

Without knowing which blanks from a production system were made for cutting tasks, and which were merely byproducts of predetermination or retouch/recycling, our ability to reconstruct the economy of hominin behavior surrounding blank cutting edge production and model changes in its efficiency are severely limited. Compounding this issue is the fact that most lithic assemblages are palimpsests, in which blanks from numerous different reduction systems are mixed and fragmented via selection for retouch, recycling, and/or transportation away from a site. The potential impact of site function and artifact transport on the frequency of blank preservation within a lithic assemblage and its influence on archaeological measures of blank cutting edge efficiency at T17 is of particular interest.

### Interpreting microblade frequency

Although we see a new blank production technology appear at T17 during MIS 2, which perhaps had a more efficient scaling of cutting edge length to mass (Fig 8), the way that the technology was utilized at the site does not seem to have significantly increased the efficiency of the assemblage it was a part of (Fig 5). However, our simulations also suggest that if microblades were discarded at T17 at a frequency seen at other MIS 2 microblade sites in East Asia, a significant increase in cutting edge efficiency would likely be observable at the assemblage level ‐ see also Kadowaki et al.’s study [18] where bladelet frequency seems to positively affect cutting edge efficiency. This could mean that microblade technologies might have indeed appeared at T17 in a context where increasing cutting edge efficiency was advantageous at the assemblage level, but preservation biases prevent us from observing this. In such a scenario where microblade production was adopted due to the fitness benefit it provided when invested in and made at a high enough frequency, how might we explain the preservation of only a handful of complete microblades within the LU2 assemblage?

One explanation may be site excavation extent. As the area excavated at T17 is currently only ca. 18 m^2^, the studied sample likely captures only a small part of the total site. The possibility of site structuring, where some spaces of the site may have been preferentially used for microblade related activities, could also mean that evidence for microblade production may be better represented in areas which have not yet been systematically excavated. Future field work at T17 will be able to better test this scenario.

A potential recovery bias of microblades at T17 may also be explained by taphonomic processes. Because of their small size, microblades may have been preferentially transported away from the site during erosional or sheet washing events. However, the preservation of microblades alongside other small artifacts including small flakes, bladelets, lithic fragments, ostrich eggshell beads, etc. suggest that the site is largely *in situ*.

Lastly, site function and preferential artifact transport are possible explanations for recovery bias of microblades at T17. Though microblades could be used in hand for cutting tasks, or as standardized interchangeable tenons for composite cutting tools used in domestic activities on site [47], they also may have been inserted into the mortises of composite hunting weapons strategically used outside of the site [27, 43, 44, 48–50]. In the latter case, these hunting weapons, and the easily transportable microblade cores used to furnish them [45–47, 50], may have been carried away from T17 during logistical hunting forays, meaning a large portion of the production, use, and discard of microblades by people living at the site may have taken place away from the excavated area [96]. Such a strategy may have been adaptive within the ecological contexts of the Eurasian Steppe during MIS 2, where animal resources for needed for subsistence are thought to have been widely distributed in part due to a downturn in environmental carrying capacity [51].

To recap, three scenarios concerning the appearance of microblades at T17, and the relationship of this event to parameters of cutting edge efficiency, could have affected our findings. In the first scenario, the lack of difference in efficiency observed at the assemblage level may indicate that the appearance of microblades at the site was not facilitated by selection pressure on increasing cutting edge production, but perhaps on other parameters of lithic efficiency and standardization [17, 27]. In the second, we posited that selection pressures may have favored the appearance of microblade technology for its cutting edge efficiency, but the assumption that fitness related changes in efficiency are only meaningful at the assemblage level limits our ability to detect them. Lastly, we considered a scenario where the appearance of microblades at T17 was facilitated by selection pressure on increasing cutting edge efficiency at the assemblage level, but preservation bias of microblades related to artifact recovery, site formation, and/or site function also prevents us from detecting this.

While we see no reason to favor any one of these three scenarios over the others, we do think their consideration can help improve future studies of lithic technological change during the Plio-Pleistocene. Investigations that make fewer assumptions about the scaling relationships of lithic blanks and how changes in blank cutting edge efficiency manifest in the archaeological record will strengthen regional studies like ours, as well as larger-scale diachronic and synchronic evolutionary studies of this critical lithic technological parameter.

## Conclusions

We adapted an approach for analyzing blank cutting edge efficiency which does not make assumptions about the scaling relationship between cutting edge length and mass, but instead discovers what their scaling is from the lithic sample under investigation. We used this approach to test if cutting edge efficiency increased within the Upper Paleolithic record of Tolbor-17 between periods before and after the introduction of microblade technology to the site. Our model found no statistical difference in cutting edge efficiency between the two assemblages, yet more conventional methods which assume a particular scaling did. A signature of improved cutting edge efficiency between the two levels is only observed within our model when microblade frequencies are artificially increased via simulation. Our results highlight the importance of properly identifying scaling relationships underlying blank cutting edge efficiency, and questions current assumptions about how changes in cutting edge efficiency are most dependably detected within the lithic archaeological record.

## Supporting information

S1 Data Metric and attribute data for blank sample (LU2 and LU3). (CSV)

S1 File Supporting information. (DOCX)

S2 File R-script for running analyses and generating figures. (R)

S1 Checklist Inclusivity in global research questionnaire. (DOCX)

## References

[1] Leakey MD. Olduvai Gorge Volume 3: Excavations in Beds I and II, 1960–1963. Cambridge University Press; 1971.

[2] Toth NP. The stone technologies of early hominids at Koobi Fora, Kenya: an experimental approach. PhD Thesis. The University of California, Berkely. 1982. Available from: https://www.proquest.com/docview/303067974

[3] Potts R. Early Hominid Activities at Olduvai: Foundations of Human Behaviour. New York: Aldine de Gruyter; 1988.

[4] McPherron SP, Alemseged Z, Marean CW, Wynn JG, Reed D, Geraads D, et al., 2010. Evidence for stone-tool-assisted consumption of animal tissues before 3.39 million years ago at Dikika, Ethiopia. Nature. doi: 10.1038/nature09248 20703305

[5] Harmand S, Lewis JE, Feibel CS, Lepre CJ, Prat S, Lenoble A, et al., 2015. 3.3-million-year-old stone tools from Lomekwi 3, West Turkana, Kenya. Nature. doi: 10.1038/nature14464 25993961

[6] Proffitt T, Luncz LV, Falótico T, Ottoni EB, de la Torre I, and Haslam M, 2016. Wild Monkeys flake stone tools. Nature. doi: 10.1038/nature20112 27760117

[7] Proffitt T, Reeves JS, Falótico T, Arroyo A, de la Torre I, Ottoni EB, et al., 2023. Identifying intentional flake production at the dawn of technology: A technological and 3D geometric morphometric study. J Arch Sci. doi: 10.1016/j.jas.2023.105740

[8] Braun DR, Aldeias V, Archer W, Arrowsmith JR, Baraki N, Campisano CJ, et al., 2019. Earliest known Oldowan artifacts at >2.58 Ma from Ledi-Geraru, Ethiopia, highlight early technological diversity. PNAS. doi: 10.1073/pnas.1820177116 31160451 PMC6575601

[9] Thompson JC, Carvalho S, Marean CW, and Alemseged Z, 2019. Origins of the Human Predatory Pattern: The Transition to Large-Animal Exploitation by Early Hominis. Curr Anthropol. doi: 10.1086/701477

[10] Plummer TW, Oliver JS, Finestone EM, Ditchfield PW, Bishop LC, Blumenthal SA, et al., 2023. Expanded geographic distribution and dietary strategies of the earliest Oldowan hominins and Paranthropus. Science. doi: 10.1126/science.abo7452 36758076

[11] Tixier J. Procédés d’analyse et questions de terminologie concernant l’étude des ensembles industriels du Paléolithique récent et de l’Epipaléo-lithique dans l’Afrique du Nord-Ouest (in French). In: Bishop WW and Desmond-Clark J, editors. Background to evolution in Africa. Chicago: Proceedings of a Symposium held at Burg Wartenstein Austria; 1967. pp. 771–820

[12] Braun DR and Harris JWK. Technological Developments in the Oldowan of Koobi Fora: Innovative Techniques of Artifact Analysis. In Moreno JM, Torcal RM, and Sainz IT, editors. Oldowan: Rather More than Smashing Stones. University of Barcelona Press, Barcelona; 2003. pp. 117–144.

[13] Tactikos JC. A re-evaluation of Palaeolithic stone tool cutting edge production rates and their implications. In: Moloney N and Shott MJ, editors. Lithic analysis at the millenium. London, U.K.: Univeristy College London Press; 2003. pp. 151–162

[14] Mackay A, 2008. A method for estimating edge length from flake dimensions: use and implications for technological change in the southern African MSA. J Archaeol Sci. doi: 10.1016/j.jas.2007.05.013

[15] Lin SC, Režek Z, Braun DR, Dibble HL, 2013. On the utility and economization of unretouched flakes: the effects of exterior platform angle and platform depth. Am Antiq. doi: 10.2307/43184970

[16] Muller A and Clarkson C, 2016. Identifying Major Transitions in the Evolution of Lithic Cutting Edge Production Rates. PLoS One. doi: 10.1371/journal.pone.0167244 27936135 PMC5147885

[17] Režek Z, Dibble HL, McPherron S.P, Braun D.R., and Lin S.C., 2018. Two million years of flaking stone and the evolutionary efficiency of stone tool technology. Nat Eco Evo. doi: 10.1038/s41559-018-0488-4 29507377

[18] Kadowaki S, Wakano JY, Tamura T, et al., 2024. Delayed increase in stone tool cutting-edge productivity at the Middle-Upper Paleolithic transition in southern Jordan. Nat Commun. doi: 10.1038/s41467-024-44798-y 38326315 PMC10850154

[19] Aiello LC and Wheeler P, 1995. The Expensive-Tissue Hypothesis: The Brain and Digestive System in Human and Primate Evolution. Curr Anthropol. doi: 10.1086/204350

[20] Antón SC, Potts R, & Aiello LC, 2014. Evolution of early Homo: An integrated biological perspective. Science. doi: 10.1126/science.1236828 24994657

[21] Grabowski M, 2016. Bigger Brains Led to Bigger Bodies? The Correlated Evolution of Human Brain and Body Size. Curr Anthropol. doi: 10.1086/685655

[22] El Zaatari S, Grine FE, Ungar PS, and Hublin J-J, 2016. Neandertal versus Modern Human Dietary Responses to Climatic Fluctuations. PLoS One. doi: 10.1371/journal.pone.0153277 27119336 PMC4847867

[23] Serangeli J, Rodríguez-Álvarez B, Tucci M, Verheijen I, Bigga G, Böhner U, et al., 2018. The Project Schoningen from an ecological and cultural perspective. Quat Sci Rev. doi: 10.1016/j.quascirev.2018.08.020

[24] Pontzer H, 2012. Ecological Energetics in Early Homo. Curr Anthropol. doi: 10.1086/667402

[25] Prasciunas MM., 2007. Bifacial Cores and Flake Production Efficiency: An Experimental Test of Technological Assumptions. Am Antiq. doi: 10.2307/40035817

[26] Eren MI, Greenspan A, and Sampson CG, 2008. Are Upper Paleolithic blade cores more productive than Middle Paleolithic discoidal cores? A replication experiment. J Hum Evol. doi: 10.1016/j.jhevol.2008.07.009 18835009

[27] Kuhn SL, and Shimelmitz R, 2023. From Hafting to Retooling: Miniaturization as Tolerance Control in Paleolithic and Neolithic Blade Production. J Archaeol Method Theory. doi: 10.1007/s10816-022-09575-5

[28] Sheets PD and Muto GR, 1972. Pressure blades and total cutting edge: an experiment in lithic technology. Science. doi: 10.1126/science.175.4022.632 17808802

[29] Gómez Coutouly YA, 2018. The emergence of Pressure Knapping Microblade Technology in Northeast Asia. Radiocarbon. doi: 10.1017/RDC.2018.30

[30] Crabtree DE, 1968. Mesoamerican Polyhedral Cores and Prismatic Blades. Am Antiq. doi: 10.2307/278596

[31] Li L, Lin SC, McPherron SP, Abdolahzadeh A, Chan A, Dogandžić T, et al., 2022. A Synthesis of the Dibble et al. Controlled Experiments into the Mechanics of Lithic Production. J Archaeol Method Theory. doi: 10.1007/s10816-022-09586-2

[32] Speth JD, 1972. Mechanical basis of percussion flaking. Am Antiq. doi: 10.2307/278884

[33] Cotterell B, Kamminga J, & Dickson FP, 1985. The essential mechanics of conchoidal flaking. Int J Fract. doi: 10.1007/BF00125471

[34] Cotterell B, & Kamminga J, 1987. The formation of flakes. Am Antiq. doi: 10.2307/281378

[35] McPherron SP, Abdolahzadeh A, Archer W, Chan A, Djakovic I, Dogandžić T, et al., 2020. Introducing platform surface interior angle (PSIA) and its role in flake formation, size and shape. PLoS One. doi: 10.1371/journal.pone.0241714 33206671 PMC7673556

[36] Lin SC, Režek Z, Abdolahzadeh A, Braun DR, Dogandžić T, Leader GM, et al., 2022. The mediating effect of platform width on the size and shape of stone flakes. PLoS One. doi: 10.1371/journal.pone.0262920 35061837 PMC8782408

[37] Dibble HL, & Režek Z, 2009. Introducing a new experimental design for controlled studies of flake formation: Results for exterior platform angle, platform depth, angle of blow, velocity, and force. J Archaeol Sci. doi: 10.1016/j.jas.2009.05.004

[38] Magnani M, Režek Z, Lin SC, Chan A, & Dibble HL, 2014. Flake variation in relation to the application of force. J Archaeol Sci. doi: 10.1016/j.jas.2014.02.029

[39] Van Peer P, 2021. A mechanical framework of conchoidal flaking and its place in lithic systematics. J Paleolit Archaeol. doi: 10.1007/s41982-021-00086-5

[40] Nakazawa Y, Izuho M, Takakura J, and Yamada S, 2005. Toward and understanding of technological variability in microblade assemblages in Hokkaido, Japan. Asian Perspect. Vol 44, No 2, pp 276–292.

[41] Takakura J, 2010. Refitted material and consideration of lithic reduction sequence among the microblade assemblages: a view from the Okushirataki-1 site, Hokkaido, Northern Japan. Asian Perspect. Vol 49, No 2, pp 332–347.

[42] Režek Z, Lin S, Iovita R, & Dibble HL, 2011. The relative effects of core surface morphology on flake shape and other attributes. J Archaeol Sci. doi: 10.1016/j.jas.2011.01.014

[43] Elston RG and Brantingham PJ. Microlithic technology in northern Asia: a risk-minimizing strategy of the late Paleolithic and early Holocene. In: Elston RG, Kuhn SL, editors. Thinking small: global perspectives on Microlithization. Virginia: Archaeological Papers of the American Anthropological Association; 2002. pp 103–116.

[44] Goebel, T. The “microblade adaptation” and recolonization of Siberia during the Late Upper Pleistocene. In: Elston RG, Kuhn SL, editors. Thinking small: global perspectives on Microlithization. Virginia: Archaeological Papers of the American Anthropological Association; 2002. pp. 117–132

[45] Yi M, Gao X, Li F, and Chen G, 2016. Rethinking the origin of microblade technology: A chronological and ecological perspective. Quat Int. doi: 10.1016/j.quaint.2015.07.009

[46] Elston RG, Dong G, and Zhang D, 2011. Late Pleistocene intensification technologies in Northern China. Quat Int. doi: 10.1016/j.quaint.2011.02.045

[47] Yi M, Barton L, Morgan C, Liu D, Chen F, Zhang Y, et al., 2013. Microblade technology and the rise of serial specialists in north-central China. J Anthropol Archaeol. doi: 10.1016/j.jaa.2013.02.001

[48] Grimaldi S, Santaniello F, Cohen DJ, Shi J, and Song Y, 2023. Last Glacial Maximum Microblade Production at Shizitan 29 and its Implications for North China Pressure Technology. J Field Archaeol. doi: 10.1080/00934690.2022.2137754

[49] Zhao C, Wang Y, Gu W, Wang S, Wu X, Gao X, et al., 2021. The emergence of early microblade technology in the hinterland of North China: a case study based on the Xishi and Dongshi site in Henan Province. Archaeol Anthropol Sci. doi: 10.1007/s12520-021-01338-9

[50] Inizan M-L, 2012. Pressure Debitage in the Old World: Forerunners, Researchers, Geopolitics–Handing on the Baton. In: Desrosiers P. (eds) The Emergence of Pressure Blade Making. Springer. doi:10.1007/978-1-4614-2003-3_2

[51] Zhu D, Ciais P, Chang J, Krinner G, Peng S, Viovy N, et al., 2018. The large mean body size of mammalian herbivores explains the productivity paradox during the Last Glacial Maximum. Nat Eco Evol. doi: 10.1038/s41559-018-0481-y 29483680 PMC5868731

[52] Bettinger RL, Barton L, Morgan C, Chen FH, Wang H, Guilderson TP, et al., 2010. The Transition to Agriculture at Dadiwan, People’s Republic of China. Curr Anthropol. doi: 10.1086/655982

[53] Zwyns N, Paine CH, Tsedendorj B, Talamo S, Fitzsimmons KE, Gantumur A, et al., 2019. The Northern Route for Human dispersal in Central and Northeast Asia: New evidence from the site of Tolbor-16, Mongolia. Sci Rep. doi: 10.1038/s41598-019-47972-1 31409814 PMC6692324

[54] Gallo G, Fyhrie M, Paine C, Ushakov SV, Izuho M, Gunchinsuren B, et al., 2021. Characterization of structural changes in modern and archaeological burnt bone: Implications for differential preservation bias. PLoS One. doi: 10.1371/journal.pone.0254529 34320009 PMC8318310

[55] Gladyshev SA, Olsen JW, Tabarev AV, and Jull AJT, 2012. The Upper Paleolithic of Mongolia: Recent finds and new perspectives. Quat Int. doi: 10.1016/j.quaint.2012.01.032

[56] Tabarev A., Gunchinsuren B., Gillam J., Gladyshev S., Dogandžić T., Zwyns N, et al., 2012. Kompleks Pamiatnikov Kamennogo Veka v Doline r.Ikh Tulberiin-Gol, Severnaya Mongolia (razvedochnye raboty s ispolzovaniem GIS-Technnologii v. 2011 g) [in Russian]. Studia Archaeol. Insit. Archaeol. Acad. Scient. Mongol. Vol. 32, pp. 26–43

[57] Gladyshev G, Jull AJT, Dogandžić T, Zwyns N, Olsen JW, Richards MP, et al., 2013. Radiocarbon dating of Paleolithic sites in the Ikh-Tulberiin-Gol River Valley, Northern Mongolia. Vestnik. 12(5): 44–48.

[58] Zwyns N, Gladyshev SA, Gunchinsuren B, Bolorbat T, Flas D, Dogandžić T, et al., 2014. The open-air site of Tolbor 16 (Northern Mongolia): Preliminary results and perspectives. Quat Int. doi: 10.1016/j.quaint.2014.05.043

[59] Rybin EP, Paine CH, Khatsenovich AM, Tsedendorj B, Talamo S, Marchenko DV, et al., 2020. A New Upper Paleolithic occupation at the site of Tolbor-21 (Mongolia): Site formation, human behavior and implications for the regional sequence. Quat Int. doi: 10.1016/j.quaint.2020.06.022

[60] Derevianko AP, Zenin AN, Rybin EP, Gladyshev SA, Tsybankov AA, Olsen JW, et al., 2007. The technology of early Upper Paleolithic reduction in Northern Mongolia: The Tolbor-4 site. Archaeol. Ethnol. Anthropol. Eurasia. doi: 10.1134/S1563011007010021

[61] Zwyns N, Gladyshev SA, Tabarev A, Gunchinsuren B, 2014. Mongolia: Paleolithic. In: Smith C. (eds) Encyclopedia of Global Archaeology. Springer, New York, NY. doi: 10.1007/978-1-4419-0465-2_1905

[62] Fu Q, Li H, Moojani P, Jay F, Slepchenko SM, Bondarev AA, et al., 2014. Genome sequence of a 45,000-year-old modern human from western Siberia. Nature. doi: 10.1038/nature13810 25341783 PMC4753769

[63] Morgan C, Barton L, Yi M, Bettinger RL, Gao X, and Peng F, 2014. Redating Shuidonggou Locality 1 and Implications for the Initial Upper Paleolithic in East Asia. Radiocarbon. doi: 10.2458/56.16270

[64] Li F, Vanwezer N, Boivin N, Gao X, Ott F, Petraglia M, et al., 2019. Heading north: Late Pleistocene environments and human dispersals in central and eastern Asia. PLoS One. doi: 10.1371/journal.pone.0216433 31141504 PMC6541242

[65] Zwyns N, and Lbova LV, 2019. The Initial Upper Paleolithic of Kamenka site, Zabaikal region (Siberia): A closer look at the blade technology. Archaeol. Res. Asia. doi: 10.1016/j.ara.2018.02.004

[66] Zwyns N, 2021. The Initial Upper Paleolithic in Central and East Asia: Blade Technology, Cultural Transmission, and Implications for Human Dispersals. J of Paleo Arch. doi: 10.1007/s41982-021-00085-6

[67] Devièse T, Massilani D, Yi S, Comeskey D, Nagel S, Nickel B, et al., 2019. Compound-specific radiocarbon dating and mitochondrial DNA analysis of the Pleistocene hominin from Salkhit Mongolia. Nat Commun. doi: 10.1038/s41467-018-08018-8 30700710 PMC6353915

[68] Dogandžić T, Braun DR, and McPherron SP, 2015. Edge Length and Surface Area of a Blank: Experimental Assessment of Measures, Size Predictions and Utility. PLoS One. doi: 10.1371/journal.pone.0133984 26332773 PMC4557928

[69] Mackay A, 2011. Nature and significance of the Howiesons Poort to post-Howiesons Poort transition at Klein Kliphuis rockshelter, South Africa. J Archaeol Sci. doi: 10.1016/j.jas.2011.02.006

[70] Will M, Bader GD, and Conard NJ, 2014. Characterizing the Late Pleistocene MSA Lithic Technology of Sibudu, KwaZulu-Natal, South Africa. PLoS One. doi: 10.1371/journal.pone.0098359 24878544 PMC4039507

[71] Conard NJ and Will M, 2015. Examining the Causes and Consequences of Short-Term Behavioral Change during the Middle Stone Age at Sibudu, South Africa. PLoS One. doi: 10.1371/journal.pone.0130001 26098694 PMC4476744

[72] Bader GD, Cable C, Lentfer C, and Conard NJ, 2016. Umbeli Belli Rock Shelter, a forgotten piece from the puzzle of the Middle Stone Age in KwaZulu-Natal, South Africa. J Archaeol Sci Rep. doi: 10.1016/j.jasrep.2016.08.038

[73] Caruana MV, Tasker D, and Stratford DJ, 2019. Identifying Raw Material Transportation and Reduction Strategies from the Lithic Scatters at Elandsdrift Farm (Cradle of Humankind World Heritage Site), South Africa. Afr Archaeol Rev. doi: 10.1007/s10437-019-09331-3

[74] Proffitt T, Reeves JS, Braun DR, Malaivjitnond S, and Luncz L, 2023. Wild macaques challenge the origin of intentional tool production. Sci Adv. doi: 10.1126/sciadv.ade8159 36897944 PMC10005173

[75] Key AJM and Lycett SJ, 2014. Edge Angle as a Variably Influential Factor in Flake Cutting Efficiency: An Experimental Investigation of its Relationship with Tool Size and Loading. Archaeometry. doi: 10.1111/arcm.12140

[76] Key AJM and Lycett SJ, 2014. Are bigger flakes always better? An experimental assessment of flake size variation on cutting efficiency and loading. J Archaeol Sci. doi: 10.1016/j.jas.2013.07.033

[77] Stout D, Rogers M., Jaeggi AV, and Semaw S, 2019. Archaeology and the Origins of Human Cumulative Culture: A Case Study from the Earliest Oldowan at Gona, Ethiopia. Curr Anthropol. doi: 10.1086/703173

[78] Morgan TJH, Uomini NT, Rendell LE, Chouinard-Thuly L, Street SE, Lewis HW, et al., 2015. Experimental evidence for the co-evolution of hominin tool-making teaching and language. Nat Commun. doi: 10.1038/ncomms7029 25585382 PMC4338549

[79] Braun DR, 2005. Examining Flake Production Strategies: Examples from the Middle Paleolithic of Southwest Asia. Lithic Technol. doi: 10.1080/01977261.2005.11721029

[80] West BJ, West D, and Kott A, 2020. Size and History Combine in Allometry Relation of Technology Systems. J. Def. Model. Simul. doi: 10.1177/1548512920942327

[81] Inizan ML, Reduron-Ballinger M, Roche H, and Tixier J. Technology and Terminology of Knapped Stone. CREP Publishing; 1999.

[82] Venditti F, Cristiani E, Nunziante-Cesaro S, Agam A, Lemorini C. and Barkai R, 2019. Animal residues found on tiny Lower Paleolithic tools reveal their use in butchery. Sci Rep. doi: 10.1038/s41598-019-49650-8 31506589 PMC6736941

[83] Ramanujan S, 1914. Modular equations and approximations to π. Quart. J. Math. XLV: 350–372

[84] Gurtov AN and Eren MI, 2014. Lower Paleolithic bipolar reduction and hominin selection of quartz at Olduvai Gorge, Tanzania: What’s the connection? Quat Int. doi: 10.1016/j.quaint.2013.08.010

[85] Putt S, 2015. The origins of stone tool reduction and the transition to knapping: An experimental approach. J Archaeol Sci Rep. doi: 10.1016/j.jasrep.2015.01.004

[86] Dibble HL and Bernard MC, 1981. A comparative study of basic edge angle measurement techniques. Am Antiq. doi: 10.2307/280156

[87] Weisberg S. Applied Linear Regression, 3^rd^ Edition. Wiley & Sons Inc., Hoboken, New Jersey; 2005.

[88] Cragg J.G., 1971. Some Statistical Models for Limited Dependent Variables with Application to the Demand for Durable Goods. Econometrica. doi: 10.2307/1909582

[89] Gauvrit Roux R., Teten’kin AV, and Henry A., 2021. Which uses for the Late Glacial microblades of Eastern Siberia? Functional analysis of the lithic assemblage of Kovrizhka IV, Level 6. Reports of the Laboratory of Ancient Technologies. doi:10.21285/2415-8739-2021-2-9-22

[90] Guan Y, Wang X, Wang F, Olsen JW, Pei S, and Gao X, 2020. Microblade remains from the Xishahe site, North China and their implications for the origin of microblade technology in Northeast Asia. Quat Int. doi: 10.1016/j.quaint.2019.03.029

[91] Nakazawa Y and Akai F, 2020. The Last Glacial Maximum Microblades from Kashiwadai 1 in Hokkaido, Japan. Lithic Technol. doi: 10.1080/01977261.2020.1734755

[92] R Core Team (2014). R: A language and environment for statistical computing. R Foundation for Statistical Computing, Vienna, Austria. URL http://www.R-project.org/

[93] Carstensen B, Plummer M, Laara E, and Hills M, 2022. Epi: A Package for Statistical Analysis in Epidemiology. R package version 2.46. URL https://CRAN.R-project.org/package=Epi

[94] Wickham H and Seidel D, 2022. _scales: Scale Functions for Visualization_. R package version 1.2.0. URL https://CRAN.R-project.org/package=scales

[95] Muller A and Clarkson C, 2022. Filling in the Blanks: Standardization of Lithic Flake Production Throughout the Stone Age. Lithic Technol. doi:10.1080/01977261.2022.2103290

[96] Gravel-Miguel C, Murray JK, Schoville BJ, Wren CD, and Marean CW, 2021. Exploring variability in lithic armature discard in the archaeological record. J Hum Evol. doi: 10.1016/j.jhevol.2021.102981 33848696

