# Supplementary material for: Analyzing blank cutting edge efficiency associated with the adoption of microblade technology: A case study from Tolbor-17, Mongolia
Source: PLoS One. 2024 Aug 16;19(8):e0305136. doi: 10.1371/journal.pone.0305136 (PMC11329150; doi:10.1371/journal.pone.0305136)
Supplement: S1 File — (DOCX) [file pone.0305136.s002.docx]

**Analyzing blank cutting edge efficiency associated with the adoption of microblade technology: a case study from Tolbor 17, Mongolia**

Corey L. JOHNSON

Tsedendorj BOLORBAT

Mark N. GROTE

Clea H. PAINE

Guunii LKHUNDEV

Davaakhuu ODSUREN

Masami IZUHO

Byambaa GUNCHINSUREN

Nicolas ZWYNS

**Supplementary Information**

1. **Assemblage demographics**
2. **Sensitivity to statistical approach**

**Assemblage demographics**

The number of blanks in the LU2 and LU3 samples and an initial technological categorization made by one of the authors (CLJ) during cutting edge length data collection can be found in the table below (Table A). Blades, bladelets, microblades, and laminar flakes - which show some evidence of being part of blade and bladelet debitage but lack some defining criteria of blades and bladelets [1] - together make up a sizable component of the LU2 (ca. 46%) and LU3 (ca. 30%) samples. Technical blanks with some connection to the production of blades, bladelets and microblades – such as core tablets, crest flakes, outrepassé flakes, debordant blanks, and decortication blanks (blade crests are included in the blade, bladelet, and microblade counts) – make up a small portion of the assemblage, particularly in LU2. Nondescript flakes are the dominate single blank category in both assemblages, with ca. 35% and 47% of all blanks falling under this category in LU2 and LU3, respectively. The low background frequency of preferential flakes such as Kombewa and bifacial thinning flakes, as well as Levallois and non-preferential pseudo-Levallois types likely represent an incidental aspect of the debitage which took place at the site.

**Table A. Number of blanks in the LU2 and LU3 samples by blank type.**

| **Blank Type** | **LU2** | **LU3** | **Total** |
| --- | --- | --- | --- |
| Laminar Flake | 30 | 64 | 94 |
| Blade | 22 | 27 | 49 |
| Bladelet | 10 | 39 | 49 |
| Microblade | 10 | 0 | 10 |
| Crest Flake | 0 | 2 | 2 |
| Core Tablet | 0 | 10 | 10 |
| Outrepassé | 3 | 5 | 8 |
| Decortication | 6 | 31 | 37 |
| Debordant | 11 | 18 | 29 |
| Nondescript Flake | 55 | 204 | 259 |
| Levallois | 2 | 7 | 9 |
| Pseudo-Levallois | 4 | 17 | 21 |
| Kombewa | 3 | 7 | 10 |
| Bifacial thinning flake | 0 | 2 | 2 |
| **Total** | 156 | 433 | **589** |

**Sensitivity to statistical approach**

To check the sensitivity of our results to different statistical methods, we contrasted direct or proxy measurements of cutting edge length, in combination with approaches that infer or impose allometry, as in a factorial experiment. The primary analysis in the Main Text uses direct measurements of blank cutting edge length in a model that infers the allometric relationship between cutting edge length and mass, to draw conclusion about differences in cutting edge efficiency between levels. For comparative purposes, the Main Text also reports analyses, based on the work of Mackay [2] and Režek et al. [3], which use proxy measure of cutting edge length and a statistical test that imposes allometric relationships. At a Reviewer’s suggestion we here generate results from the remaining factorial combinations: 1) proxied measures of cutting edge length with inferred allometry; and 2) direct measures of cutting edge length with imposed allometry. Although the proxy measurements can be made even when there is no useful segment of cutting edge length, for standardization purposes these follow up analyses were performed on the same sample used in the Main Text (i.e., only blanks with more the 0mm of cutting edge length).

First, we employ a regression model as described by equations 1 and 2 in the Material and Methods section of the Main Text, though substituting the proxy measurements for perimeter length outlined by Mackay [2] and Režek et al. [3] in place of direct measurement of cutting edge length (Fig A).

When the Mackay proxy is used (Fig A.A) there is a slight separation between the confidence bands for the two levels, which can be observed for the smallest blanks, including some microblades (i.e., those blanks less than ca. 0.03g based on the log10 scale). This suggests a difference in cutting edge efficiency between the two levels for blanks of this size.

When the Režek et al. proxy is used (Fig A.B) no difference between the levels is observable, as for the primary analysis shown in Fig 5 of the Main Text.


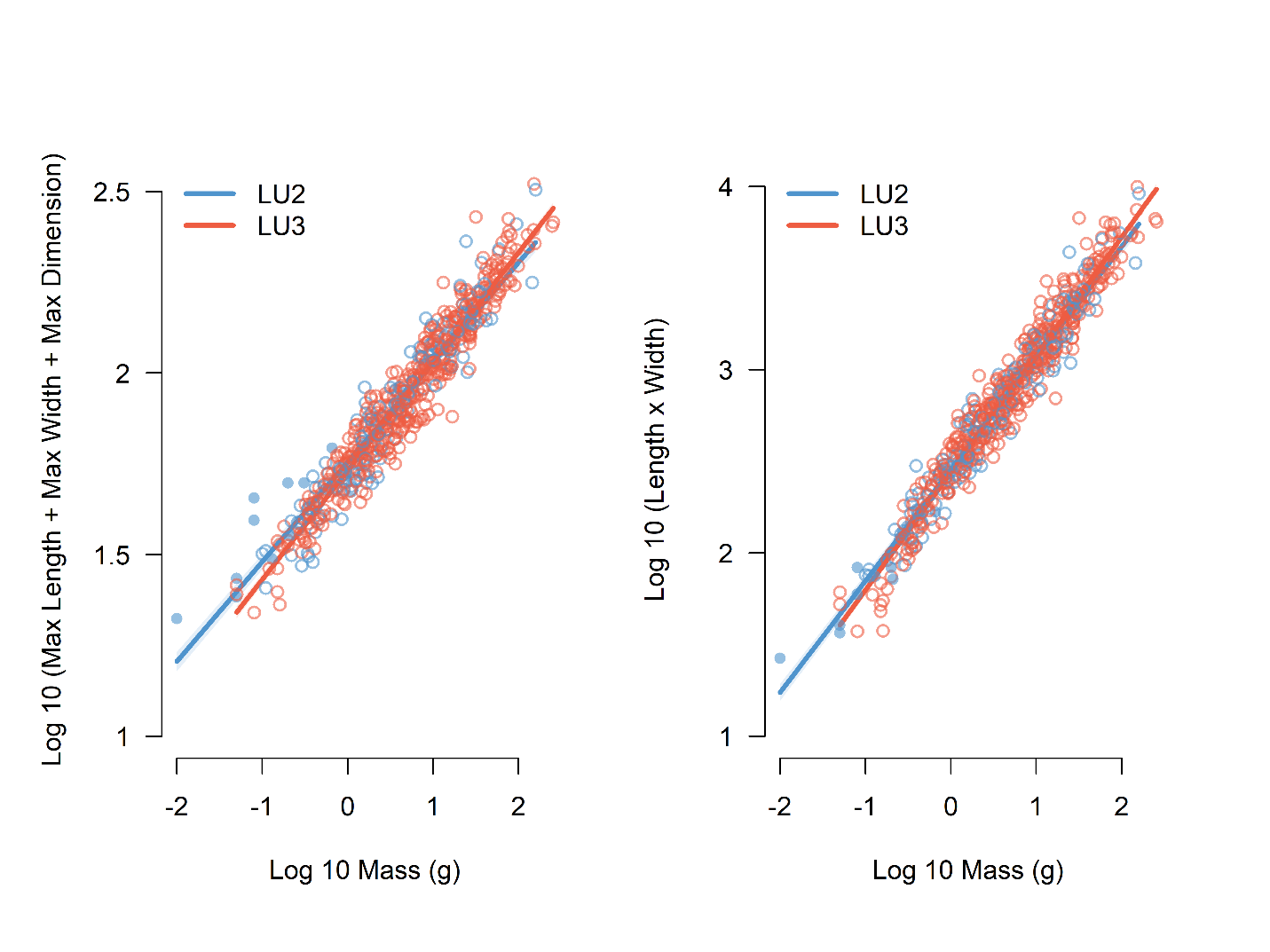


A)

B)

**Fig A. Graphical display of the fitted model comparing all complete blanks from LU3 and LU2 using proxy measurement data for cutting edge length. A)** Mackay [2] cutting edge length estimate; **B)** Režek et al. [3] cutting edge length estimate. Microblades from LU2 are indicated by closed circles (●).

Moving on to methods that use direct measures of cutting edge length with imposed allometry, we used the blank cutting edge length observations reported in the Main Text in the numerator of the Mackay [2] and Režek et al [3] equations, respectively, accepting the implied forms of allometry. We apply Wilcoxon rank sum tests in both cases for comparability (Fig B and C). In both cases significant differences between the levels are suggested (Mackay: W = 40146, p < 0.001; Režek et al.: W = 40775, p < 0.001) echoing results obtained when using the methods as originally described (Fig 6 and 7 in the Main Text).


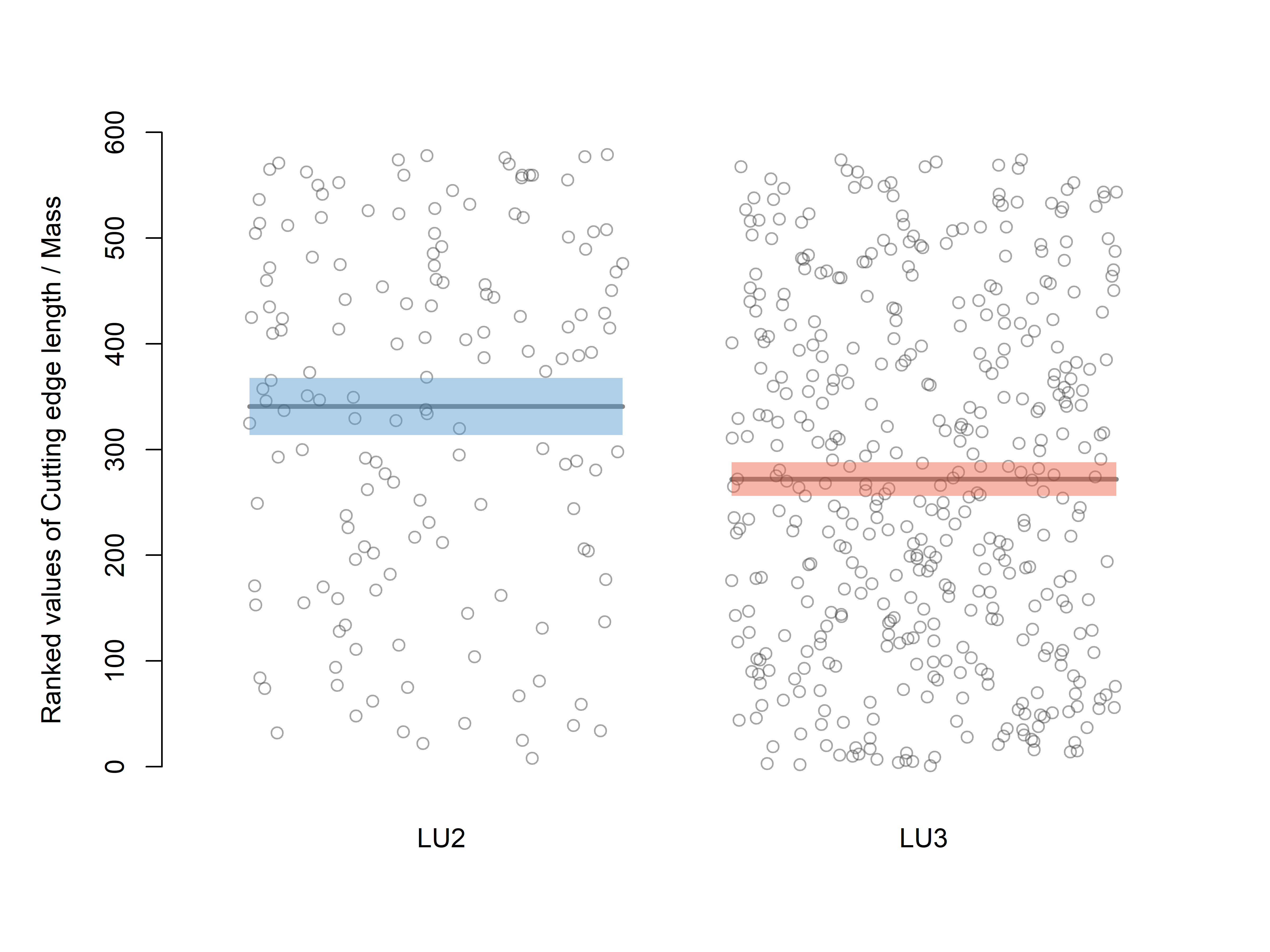


**Fig B. Graphical display of ranked values of the cutting edge efficiency statistic in Mackay [2], substituting direct measures of cutting edge length for proxy measures.** Means of ranked values are shown as horizontal lines, with shaded bands indicating two standard-error intervals around the means. Means and standard errors are shown here only for visualization purposes and do not reflect the calculation of the Wilcoxon statistic explicitly.


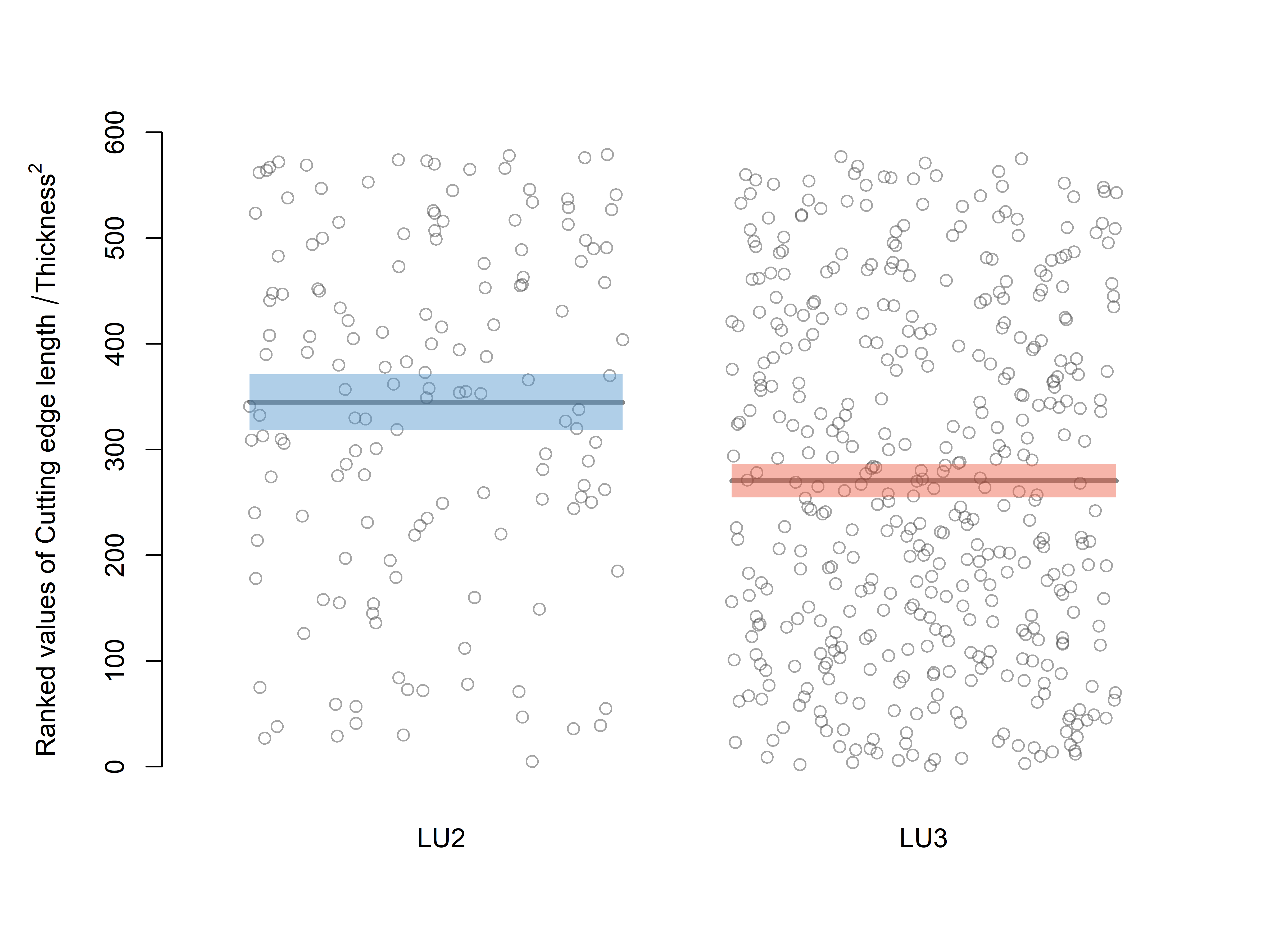


**Fig C. Graphical display of ranked values of the cutting edge efficiency statistic in Režek et al [3], substituting direct measures of cutting edge length for proxy measures.** Means of ranked values are shown as horizontal lines, with shaded bands indicating two standard-error intervals around the means. Means and standard errors are shown here only for visualization purposes and do not reflect the calculation of the Wilcoxon statistic explicitly.

**Bibliography**

1. Inizan ML, Reduron-Ballinger M, Roche H, and Tixier J. Technology and Terminology of Knapped Stone. CREP Publishing; 1999.
2. Mackay A, 2008. A method for estimating edge length from flake dimensions: use and implications for technological change in the southern African MSA. J Archaeol Sci. doi:10.1016/j.jas.2007.05.013
3. Režek Z, Dibble HL, McPherron S.P., Braun D.R., and Lin S.C., 2018. Two million years of flaking stone and the evolutionary efficiency of stone tool technology. Nat Ecol and Evol. doi:10.1038/s41559-018-0488-4
